# Assessing the Temperamental Basis of the Sense of Humor: Adaptation of the English Language Version of the State-Trait Cheerfulness Inventory Long and Standard Form

**DOI:** 10.3389/fpsyg.2018.02255

**Published:** 2018-11-27

**Authors:** Jennifer Hofmann, Hugo Carretero-Dios, Amy Carrell

**Affiliations:** ^1^Department of Psychology, University of Zurich, Zurich, Switzerland; ^2^Department of Research Methods in Behavioral Sciences, University of Granada, Granada, Spain; ^3^Department of English, University of Central Oklahoma, Edmond, OK, United States

**Keywords:** bad mood, cheerfulness, humor, sense of humor, seriousness, STCI

## Abstract

The State-Trait Model of Cheerfulness assesses the temperamental basis of the sense of humor with the traits and respective states of cheerfulness, seriousness, and bad mood. Cheerfulness is a dominant factor in current measures of the sense of humor and explains both, the disposition to engaging in smiling and laughter, as well as humor behaviors, and trait seriousness and bad mood are antagonistic to the elicitation of amusement (albeit for different reasons). Several studies have shown the validity and reliability of the STCI questionnaire in German and other language versions (i.e., Spanish). In this study, the English language version with 106 items (STCI-T <106>) was translated, checked for its item and scale characteristics, and tested with a confirmatory factor analysis approach (*N* = 1101) to investigate the factorial validity of the STCI-T <106> scale. Results show good psychometric characteristics, good internal consistencies, and a fit to the postulated underlying structure of the STCI-T. Then, the standard form with 60 items (STCI-T <60>) was developed and the psychometric characteristics initially tested. In an independent sample (*N* = 169), the characteristics of the standard form were compared to the parent form and German equivalent. It showed good psychometric characteristics, internal consistencies, as well as a good self- and peer-report congruence. To conclude, the STCI-T <106> is the measure of choice for the assessment of the temperamental basis of the sense of humor and the separate facets of the traits, while the standard form (60 items) allows of an economic assessment of cheerfulness, seriousness, and bad mood, free of context-saturated items and humor preferences.

Ruch and colleagues found intra- as well as inter-individual differences in humor-related behaviors, thoughts, motivation, and responses (Ruch, 1993; Ruch et al., 1996, 1997; Ruch and Köhler, 2007). In particular, they found that individuals differ habitually in the likelihood of engaging in humor, the frequency and intensity of amusement responses, the quality and quantity of humor production, and the appreciation of humorous interactions (for an overview, see Ruch and Hofmann, 2012). High scorers of those habitual differences are usually nominated to have a good “sense of humor.” When looking at individuals with a “good sense of humor” more closely, the authors also observed that variations in the individuals' readiness to engage in humor occurred across situations and time, indicating that there might be humor-related *states* that enhance or lower ones threshold for amusement (for an overview see Ruch and Hofmann, 2012). To explain these inter- and intra-individual differences, Ruch et al. (1996) put forward a model defining the temperamental basis of the sense of humor: the State-Trait Model of Cheerfulness.

The State-Trait Model of Cheerfulness is a multidimensional model that assesses the affective and cognitive foundation of the sense of humor, assuming this foundation to have trait-like qualities. Therefore, the model is largely free of specific contents and preferences for certain humor materials (such as “dark humor” or “nonsense humor,” certain comedians or comedy formats) but rather describes the underlying traits that predispose individuals to humor (or “humorlessness”). To account for intra-individual differences as well, the same concepts were used as traits and states, which allows for the study of mood states and their influence on humor elicitation (Ruch, 1993; Ruch et al., 1996, 1997; Ruch and Köhler, 2007; Ruch and Hofmann, 2012). The three relevant states and traits are: state and trait cheerfulness, state and trait seriousness and state and trait bad mood. While cheerfulness lowers the threshold for amusement, the latter two dimensions heighten the threshold for amusement (Ruch et al., 1996).

Cheerfulness goes along with a low threshold for amusement and liking to engage in humorous interactions (Ruch et al., 1996). Whereas both seriousness and bad mood may be perceived as forms of humorlessness (cf. McGhee, 1996), they do indeed heighten the threshold for amusement, but they are not the opposite of it. In fact, there are forms of humor that require certain degrees of seriousness (i.e., the ability to laugh at oneself, McGhee (1996) or “Ernstheiterkeit,” see Proyer and Rodden, 2013) or bad mood (e.g., cynicism; see Ruch et al., 1996). In the case of seriousness, there is lowered interest in humor and playfulness; i.e., more volition is needed for individuals to switch into a playful frame of mind or engage humorously. In the case of bad mood, negative affective states are predominant and hinder the elicitation of amusement. Within trait bad mood reasons for humorlessness differ as well: Whereas ill-humornedness may also lead to not wanting to engage in humor, sadness may lead to an inability to engage in humor (e.g., Ruch and Hofmann, 2012).

## Measurement of the state-trait cheerfulness model

The aim was to create a questionnaire largely free of content saturated items and specific humor materials/stimuli categories (Ruch et al., 1996, 1997), as this lowers the generalizability and adequate use of the scale (i.e., item judgments might be biased by the fact that certain age or cultural groups do or do not know certain materials or contents; a former criticism toward many questionnaires attempting to assess the “sense of humor,” cf. Ruch, 2007). The original German trait long form the State-Trait Cheerfulness Inventory (STCI-T <106>) provides scores for the three traits of cheerfulness (STCI-T CH; 38 items), seriousness (STCI-T SE; 37 items), and bad mood (STCI-T BM; 31 items), as well as a separate analysis of the facets of each trait. For cheerfulness, five inter-correlated facets were derived: a prevalence of cheerful mood (CH1), a low threshold for smiling and laughter (CH2), a composed view of adverse life circumstances (CH3), a broad range of active elicitors of cheerfulness and smiling or laughter (CH4), and a generally cheerful interaction style (CH5; Ruch et al., 1996). Trait bad mood (BM) is composed of the predominance of three mood states and their respective behaviors: generally being in a bad mood (BM1), sadness (i.e., despondent and distressed mood; BM2), and ill-humoredness (i.e., sullen and grumpy or grouchy feelings; BM4). Two further facets are specifically related to the sad (BM3) and ill-humored (BM5) individual's behavior in cheerfulness evoking situations, their attitudes toward such situations and the objects, persons, and roles involved (Ruch et al., 1996). Trait seriousness consists of six inter-correlated facets: the prevalence of serious states (SE1), a perception of even everyday happenings as important and considering them thoroughly and intensively (SE2), the tendency to plan ahead and set long-range goals (SE3), the tendency to prefer activities for which concrete, rational reasons can be produced (SE4), the preference for a sober, object-oriented communication style (SE5), and a “humorless” attitude about cheerfulness-related behavior, roles, persons, stimuli, situations, and actions (SE6; see Ruch et al., 1996).

The STCI-T <106> long form was constructed for three reasons: to provide an assessment of the facets, to be able to test hypotheses that link to the facets, as well as to empirically evaluate the facet model. A rational-theoretical construction strategy was applied, with the facet model serving as the basis for the generation of items (see Ruch et al., 1996). The 106 items were chosen from a pool and designed to be (1) short and understandable, (2) of diverse content, (3) covering the construct-related behavior and attitudes comprehensively, (4) be free of extreme levels of social desirability, (5) suitable for adolescents and adults, and (6) not biased toward particular populations and (7) the items needed to be logically related to the target constructs but not overlap with similar but irrelevant constructs (Ruch et al., 1996). The questionnaire utilizes a four-point answering format.

Because of the antithetical nature of the three traits (i.e., cheerfulness denominating a lowered threshold for amusement, whereas seriousness and bad mood go along with a heightened threshold for amusement), most items were positively poled, as negatively poled items could be viewed as a (positive) indicator for another trait (Ruch et al., 1996, 1997). Ruch et al. (1996) confirmed the facet model by means of factor analyses and reported mostly satisfactory to high internal consistencies for the facets (α = 0.64 to α = 0.91) and high internal consistencies for the trait total scores (CH α = 0.93, SE α = 0.91, BM α = 0.93).

To arrive at a questionnaire that is more economic for the use in research and practice, a standard trait form with 60 items was derived from the STCI-T <106>. This version is not considered for scoring facets but only total scores for the three traits of cheerfulness, seriousness, and bad mood are derived. In general, a concept-guided strategy in item reduction was coupled with an empirically guided selection of items (Ruch et al., 1996, 1997). The following criteria were considered (Ruch et al., p. 316): (a) the best corrected item to total correlation (CITC), (b) consideration of items content and representation of items of all facets, (c) roughly equal representation of the facets (where this was not possible, core facets got more weight), and (d) avoidance of very similar items as regards to content or linguistic usage (Ruch et al., 1996, p. 316). Ruch and Köhler (2007) reported high internal consistencies for the traits (CH α = 0.93, SE α = 0.88, and BM α = 0.94; *N* = 600) and the one-month retest-stability was high for the traits (between 0.77 and 0.86), in line with the expectations (Ruch et al., 1997). The three-factor structure was replicable and showed to be generalizable across samples of different nationalities and language groups.

The *state* version of the STCI initially consisted of 45 items, cheerfulness, seriousness, and bad mood as actual feeling states. To capture the mood quality, items were included that allow for a sensitive assessment of mood alternations (Ruch et al., 1997). Different facets of cheerfulness as mood states were distinguished (Ruch et al., 1997): A cheerful mood (tranquil, composed) and hilarity (more shallow, outward; Ruch et al., 1997). In state seriousness, soberness, pensiveness, and earnestness were differentiated and in state bad mood, melancholy and ill-humor were distinguished. There is also a four-point answer format, like in the trait version (Ruch et al., 1997). In an iterative process spanning over several samples, the scale was finally reduced to consist of ten items for each scale. Ruch et al. (1997) report satisfactory internal consistencies (alpha coefficients from α = 0.85 to α = 0.94) and low test-retest correlation in line with the expectations.

### Language adaptations, peer and childrens' versions

Since the first publication of the STCI-T in 1996 and the STCI-S in 1997 (Ruch et al., 1996, 1997) several translations into different languages and adaptations to other target groups (other than self-reports) were done. Table 1 gives an overview on the available versions (adapted from Ruch and Hofmann, 2012).

**Table 1:** Overview of the different versions of the STCI-T and STCI-S (adapted from Ruch and Hofmann, 2012).

**Version**	**Target**	**Facet structure**	**Language**
**TRAIT**			
STHI-T <106>	Self, peer	5 facets for cheerfulness (38 items), 6 facets for seriousness (37 items), 5 facets for bad mood (31 items)	German, English
STHI-T <104>		5 facets for cheerfulness (38 items), 6 facets for seriousness (37 items), 5 facets for bad moods (29 items)	Spanish
STHI-T <60>	Self, peer, workplace	1 score for each trait (20 items each)	Chinese (Hong Kong; Mainland China), English, French (Québec), German, Hebrew, Italian, Japanese Polish, Romanian, Russian, Slovene, Spanish (Chile)
STHI-T <30>	Self, peer	1 score for each trait (10 items each)	German, English, Italian
STHI-T <30> children	Self, peer, parent, teacher	1 score for each trait (10 items each); items adapted for children	German, Spanish
**STATE**			
STHI-S <45>	Self		German, Spanish^a^
STHI-S <30>	Self	1 score for each state (10 items each)	German, English
STHI-S <20>	Self	1 score for each state (8 items for cheerfulness, 6 seriousness, 6 bad mood)	English
STHI-S <18>	Self	1 score for each state (6 items each)	German, English, Hebrew
STHI-S <20> children	Self, peer	1 score each state (8 items for cheerfulness, 6 seriousness, 6 bad mood)	German

*Further information on the different versions and authors involved in translation and adaptation can be obtained from the authors*.

a *López-Benítez et al. (2017c)*.

As Table 1 shows, the STCI exists in over ten languages and can be applied in various settings, with various versions for self- and peer-reports (e.g., general peer-report, peer-report for parents, peer-report for the workplace; see Table 1). The instruments typically yielded comparable psychometric characteristics and correlational patterns (see Ruch and Hofmann, 2012).

### Correlations among the traits and states

Cheerfulness and bad mood are affective concepts with an antagonistic valence, supposedly leading to a negative correlation between the two. Seriousness is also a factor increasing the threshold for humor, though not on an affective level, but on the level of cognition: Seriousness refers to a frame of mind (cf. Ruch et al., 1996). Thus, correlations of seriousness to cheerfulness should be negative, but weaker as compared to bad mood, as the latter is conceptually closer, as it also refers to an affective concept. Seriousness and bad mood should be correlated positively, as they both refer to concepts potentially hindering the induction of amusement or the engagement with humor (cf. Ruch et al., 1996). For both, the STCI-T <106>, as well as the STCI-T <60> and the standard form of the state STCI-S <30> questionnaire, the results showed that homologous states and traits are separable and correlations between the converging states and traits were expectedly positive, correlations with heterologous concepts were lower (for an overview, see Ruch and Hofmann, 2012). Cheerfulness in state and trait was negatively related to trait and state seriousness and trait and state bad mood (and the latter two were positively correlated themselves). In line with the expectations, correlations among the three traits were numerically lower than among the three states (e.g., Ruch and Hofmann, 2012).

## Validation

### Trait

With respect to the *factorial* validity of the trait form, factor analyses of the facet model of the STCI-T <106> trait version supported the model by Ruch et al. (1996) with three correlated higher order factors and their five to six facets in the German version of the questionnaires, as well as the Spanish version with 104 items (Carretero-Dios et al., 2011, 2014). For the STCI-T <60> and STCI-S <30>, typically a three-factor structure could be confirmed (e.g., Ruch et al., 1996, 1997; Tapia-Villanueva et al., 2014; Chen et al., 2017).

With respect to the *convergent* and *discriminant* validity, Carretero-Dios et al. (2011) applied a multi-trait multi-method method approach (MTMM) to data of the STCI. The MTMM approach allows for the separation of different sources of individual differences (influences due to trait, method, error components). By means of confirmatory factor analysis, the convergent validity (self-reports, peer-reports, aggregated states) and discriminant validity (relationships among cheerfulness, seriousness, and bad mood) of the trait form of STCI-T <104> were tested (the Spanish version contains 104, not 106 items; see Carretero-Dios et al., 2011) and confirmed: cheerfulness, seriousness and bad mood, as both state and traits are homogeneous factors (across self reports and peer-reports). Also, aggregated states measures were correlated to their traits and these correlations were higher than for single state measures, in line with the expectations (Carretero-Dios et al., 2011). Finally, the expected patterns of correlations between the three dimensions were confirmed and the data provided support for the hypothesis that traits represent the dispositions for their respective states. Furthermore, Table 2 shows correlations of the STCI cheerfulness scale to relevant measures assessing aspects of the sense of humor (convergent validity; i.e., Ruch et al., 2011) as well as indicators of *predictive* validity (e.g., Ruch, 1997, Table 2 is adapted and updated from Ruch and Hofmann, 2012).

**Table 2:** State and trait cheerfulness and the experimental induction of amusement and cheerful mood (adapted from Ruch and Hofmann, 2012).

**Individuals high in trait cheerfulness (compared to individuals low in trait cheerfulness)**
…laugh more often and have higher increases in state cheerfulness after inhaling nitrous oxide (Ruch and Stevens, 1995)
…stay in a cheerful mood when having to elaborate proverbs with negative, misanthropic contents (Wancke, 1996)
…show facial signs of exhilaration more frequent and intense, when interacting with a clowning experimenter for 10 min (Ruch, 1997)
…have higher increases in cheerfulness after listening to funny tapes (in comparisons to tapes containing neutral contents; Ruch, 1997)
…keep a cheerful state, even when having to sit in a depressing room while working on several tasks (Ruch and Köhler, 1999)
…show more smiling and laughter (higher contraction of the zygomatic major muscle) when looking at video clips of simple news or news speaker's slips of the tongues (Beyler, 1999)
…report higher state cheerfulness, and no more physical symptoms, even when facing negative life events and stress (Hausser, 1999; Ruch and Köhler, 1999; Ruch and Zweyer, 2001)
…report using humor as a coping strategy (Ruch and Zweyer, 2001)
…have a higher pain tolerance (in the cold pressure test) after watching a funny film and producing humor to it, or smiling and laughing voluntarily at it (Zweyer et al., 2004)
…have higher rises in state cheerfulness after consuming kava extract (Thompson et al., 2004)
…report more emotional intelligence (Yip and Martin, 2006^*^)
…display BOLD activation in the inferior parietal lobule of the right hemisphere. This might be associated with a general readiness/tendency to be amused by jokes. Regions previously shown to be activated in humor appreciation studies seem more likely to be related to the understanding of individual jokes and the momentary emotion and the momentary emotional reaction of exhilaration (Rapp et al., 2008)
…score higher on all of the sense of humor facets measured by the SHS (Ruch and Carrell, 1997^*^; Ruch et al., 2009) …report less fear of being laughed at (gelotophobia, Ruch et al., 2009)
…report less need for structure (Hodson et al., 2010)
…are higher in socially warm, competent, earthy humor of the HBQD (Ruch et al., 2011)
…report more humor behavior (Ruch et al., 2011)
…show more Duchenne smiling in response to seeing distorted photographs of themselves (Beermann and Ruch, 2011)
…are higher in affiliative and self-enhancing humor styles, report less self-defeating humor of the HSQ (Martin et al., 2003; Ruch et al., 2011)
…experience an increase in state cheerfulness and show more facial displays of joy when watching funny videos alone or with a virtual companion (Hofmann et al., 2015)
…respond with more positive emotions to a clinic clowning intervention (Auerbach, 2017)
…are more sensitive to the emotional environment (López-Benítez et al., 2017a,b) …report more resilience, mindfulness, optimism, well-being and less stress (Lau et al., 2018; Hofmann et al., in press)

*Studies are presented ordered by date of publication*.

* *These studies used the pilot version of the English STCI basing on its initial translation from German*.

To summarize, Table 2 shows that trait cheerfulness correlates positively to convergent measures of the sense of humor (e.g., Ruch et al., 2011). For example, trait cheerfulness correlates positively to *coping humor* (measured by the Situational Humor Response Questionnaire, SHRQ, Martin and Lefcourt, 1984; or the Coping Humor Scale, CHS, Martin and Lefcourt, 1983), *humor styles* (measures by the Humor Styles Questionnaire, HSQ, Martin et al., 2003), the *facets of the sense of humor* (Sense of Humor Scale, SHS, McGhee, 1996), and *styles of everyday humor conduct* (e.g., Humorous Behavior Q-Sort Deck, HBQD, Craik et al., 1996; and the HUMOR, Manke, 2007). With respect to the *predictive validity*, a range of studies have shown the power of trait and state cheerfulness in the prediction of responses to humor and amusement eliciting stimuli (see Table 2). Table 2 shows the influence of state and trait cheerfulness on the experimental induction of amusement and external criteria (i.e., pain tolerance, see Zweyer et al., 2004; for a more detailed discussion, please see the original sources named in Table 2).

For seriousness and bad mood, sound correlations to convergent and discriminant measures could be established too. For example, trait bad mood and trait seriousness go along with gelotophobia (Ruch et al., 2009), less socially warm humor, and less competent humor in the HBQD, and less affiliative, self-enhancing humor in the HSQ (see Ruch et al., 2011). Moreover, trait bad mood with using less benevolent humor (that bases on a non-judgmental, cheerful outlook on the world; Hofmann et al., in press), and trait seriousness with less use of aggressive humor in the HSQ (see Ruch et al., 2011), as well as correlating negatively to most global assessments of playfulness, as well as playfulness facets (Proyer and Rodden, 2013).

Also for trait seriousness and bad mood, results on predictive validity and further aspects are in line with the expectations. For example, low trait seriousness was found to predict greater substance use, indicating that taking a less serious outlook on life also goes along with a more liberal attitude with respect to substance use (and maybe health related habits in general, see Edwards, 2012). Moreover, trait cheerfulness correlated positively with the wittiness of punch lines in a humor production task, whereas trait bad mood correlated negatively (Ruch et al., 2009). Interestingly, the numerically highest correlations were found for traits seriousness, both with the quality, as well as the quantity of humor production (with negative correlations see Ruch et al., 2009). Therefore, low seriousness predicts quantitative and qualitative aspects of humor production. With respect to positive emotional responses to situations where laughing at oneself is possible, trait cheerfulness predicted greater frequency and intensity of smiles in response to ones funnily distorted photo, whereas negative correlations of the frequency and intensity of smiling to trait bad mood were found (see Beermann and Ruch, 2011).

Looking at general evaluations of one's life (well-being, stress) as well as the dispositions of resilience and optimism, results of a recent study showed that trait cheerfulness correlates positively with resilience, optimism and well-being, whereas being negatively correlated to stress. For trait bad mood, the exact opposite pattern was found. For trait seriousness, a positive correlation to resilience was reported (Lau et al., 2018).

### State

A range of studies has undertaken the assessment of the three states of cheerfulness, seriousness and bad mood including mood changes due to natural phenomena (e.g., weather), experimental variations (e.g., experimenter personality or social behavior) and chemical substances (i.e., inhalation of “laughing gas” or “kava-kava” extract; see Ruch and Hofmann, 2012 for an overview). The results showed that one's current mood indeed alters the threshold for amusement and the manipulation of mood states might heighten or lower this threshold. For example, cheerfulness decreased after being exposed to situations inducing bad mood and was high when assessing female visitors of a carnival event (Ruch et al., 1997; Ruch and Köhler, 1999). Seriousness increased when being confronted with a 2 h mental work task, when listening to audiotapes of a serious (but also bad mood) quality and decreased in some cheerful situations, such as carnival and due to humor trainings (e.g., Falkenberg et al., 2011a,b; Ruch et al., 2018). Bad mood increased when being exposed to an adverse room environment (Ruch, 1997) and decreased after watching funny films (Hofmann et al., 2015), the inhalation of nitrous oxide (Ruch and Stevens, 1995), and sessions of humor trainings (Ruch et al., 2018). Furthermore, it was shown that that the STCI-S is a sensitive instrument for assessing longer lasting states too: As expected, depressive patients were shown to be lower in state cheerfulness and higher in state seriousness and state bad mood in comparison to the construction sample, and similarly for schizophrenic patients compared to the construction sample (Krantzhoff and Hirsch, 2001; Hirsch et al., 2010; Falkenberg et al., 2011a; Ruch et al., 2011; on depressed patients and on schizophrenic patients by Falkenberg et al., 2007).

Recently, the state-trait cheerfulness influence on self-reported disease activity levels in rheumatoid arthritis patients (Delgado-Domínguez et al., 2016) was investigated in a cross-sectional study. State cheerfulness and trait cheerfulness were assessed at the same time as a blood sample was taken from patients in order to analyze the corresponding biochemical parameters (Erythrocyte sedimentation rate and C-reactive protein), and just before measuring patient-reported disease activity. Higher state cheerfulness was observed in rheumatoid arthritis patients with lower scores in self-reported disease activity. Moreover, higher state cheerfulness was associated with lower values of C-reactive protein. Finally, results showed that the relationship between the biochemical parameters of rheumatoid arthritis and patient-reported disease activity partially depended (i.e., mediation analysis) on cheerful mood at the moment of assessment (Delgado-Domínguez et al., 2016).

### Aims of the current study

Although the STCI-T questionnaire has been used in the original German language versions and has been adapted to different languages (e.g., Carretero-Dios et al., 2014; Chen et al., 2017), an English language version, both in the long trait form with 106 items and an economic version with 60 items has not been tested and validated for research and practice. Therefore, the aim of the current study was 2-fold: Firstly, a long form with 106 items was translated, adapted, and initially validated. Secondly, the more economic short form with 60 items was adapted and initially tested (as well as being tested in an independent sample, including self-and peer-reports).

## Methods

### Participants

#### Construction sample

The sample consisted of 1,101 English speaking adults (36.2% men, 56.1% women and 7.7% indicating no gender) aged from 15 to 70 years (*M* = 24.85, *SD* = 10.11) from four different universities.

#### Replication sample

The sample consisted of 85 English speaking adults (71.1% female, 24.4% male, and 4.4% not indicating their gender), age from 18 to 78 years (*M* = 43.05, *SD* = 14.33).

#### Peer-report sample

For the *Replication Sample*, a sample of peer-raters was collected, consisting of 84 individuals (69% female, 19% male, 12% not indicting their gender), with ages ranging from 18 to 67 (*M* = 53.82, *SD* = 14.68). On average, the peer-raters spent *M* = 41.40 h with the person they had rated and they indicated that they were very familiar with the rated person (*M* = 6.40, *SD* = 1.01, Min = 4.00, Max = 7.00; scale ranging from 1 to 7).

### Instruments

#### STCI-T <106>

Cheerfulness (CH), seriousness (SE), and bad mood (BM) were assessed by the English language version of the *State-Trait-Cheerfulness-Inventory* (STCI; Ruch et al., 1996). The facet version of the STCI-T with 106 items was utilized to measure the three respective traits (and their respective facets) on a four-point scale ranging from 1 = *strongly disagree* to 4 = *strongly agree*. “I am often in a joyous mood” is an indicator for CH, “I am a rather sad person” an indicator for BM, and “one of my principles is: first work, then play” an indicator for SE. Because of the antithetical nature of the concepts a negatively keyed cheerfulness item, for example, could also be seen prototypical for seriousness or bad mood. Whereas the sentence “I feel like laughing” might indicate cheerfulness, its negation “I don't feel like laughing” might well indicate sadness. Therefore, negations were only used when they represented standing expressions used in everyday language (cf. Ruch et al., 1996).

#### STCI-T <60>

Cheerfulness (CH), seriousness (SE), and bad mood (BM) were assessed by the *State-Trait-Cheerfulness-Inventory* standard short form and peer-report form (Ruch et al., 1996). The STCI-T <60> self- and peer-report measure the respective traits (60 items) on a four-point scale ranging from 1 = *strongly disagree* to 4 = *strongly agree*.

### Procedure

#### Translation procedure

In step 1, all 106 items were translated into English by two persons (experts of humor) independently. Step 2 included a comparison of both translations, discussions about linguistic peculiarities and the intent of several items, and ended in a first list of suitable translations (coordinated by the senior author of the scale; see also Ruch and Carrell, 1997). In step 3 this list was sent to two American researchers (experts of humor) who checked it for orthographical and/or grammatical errors. Their corrections were checked for correspondence regarding the items' content and retained to a large extent. In step 4, this modified list was discussed by further two American researchers and the senior author of the original scale (WR), all familiar with the State-Trait Model of Cheerfulness. This resulted in the pilot version that was used for the current study. This procedure ensured a high level of expertise in humor research and sensitivity of the translators for the challenges of measuring humor (i.e., use of negations on an item level may make items indicative of other traits than the target trait, etc.).

#### Participant recruitment construction sample

Participants were recruited over various channels at four universities and were given a paper-pencil version of the STCI-T <106>. They returned it after completion at home or testing in the class room. After handing in the completed questionnaire, participants were thanked for their participation.

#### Participant recruitment replication sample and peer-report sample

Participants were recruited over various channels, including universities and were given a paper-pencil version of the STCI-T <60>. They returned it after completion at home or testing in the class room. All participants were encouraged to give a peer-version of the STCI-T <60> to a good friend or relative. After handing in the completed questionnaire (self-report) participants were thanked for their participation. Peer reports could be sent back via post.

All procedures complied with the ethical guidelines of the local ethics committee at the University of Zurich, Faculty of Philosophy, Department of Psychology. All participants took part in the study voluntarily and could refrain from participation at any time without any consequences to them and consent was obtained by virtue of survey completion. The anonymity of participants was ensured. An ethics approval as per institutional and national guidelines was not required.

## Results

### Psychometric characteristics of the STCI-T <106> in english

Means, standard deviations, skewness, and kurtosis of the facets and total scores are given in Table 3 (all analyses conducted in SPSS 25). Also, the internal consistencies (Cronbach Alpha; α), mean CITC for each facet and the total scores, as well as means, standard deviations, Cronbach Alpha and mean CITC of the original German scale are reported in Table 3.

**Table 3:** Descriptive statistics, corrected item to total correlation and reliability of the scales and facets of the STCI-T <106> in the construction sample.

									**German construction sample**				
	***Ni***	***M***	***Md***	***SD***	***Sk***	***K***	***M* CITC**	**α**	**α**	***M***	***SD***	***M* CITC**	***t (1100)***
CH1	8.00	24.09	24.00	4.68	−0.45	0.01	0.66	0.88	0.91	24.67	4.69	0.72	−4.12*
CH2	5.00	16.51	17.00	2.85	−0.66	0.01	0.55	0.76	0.76	15.73	2.77	0.54	9.04*
CH3	8.00	22.57	23.00	3.24	−0.03	0.15	0.34	0.65	0.76	23.96	3.84	0.46	−14.27*
CH4	8.00	26.14	26.00	3.55	−0.32	−0.21	0.43	0.72	0.68	24.29	3.74	0.38	17.35*
CH5	9.00	29.84	30.00	4.01	−0.44	−0.06	0.52	0.82	0.84	28.54	4.21	0.54	10.73*
CH Total	38.00	119.15	120.00	15.69	−0.33	−0.08	0.50	0.89	0.93	117.2	15.9	0.52	4.11*
SE1	6.00	14.21	14.00	2.84	−0.03	0.08	0.32	0.57	0.65	15.27	3.05	0.39	−12.37*
SE2	7.00	17.42	17.00	3.47	0.03	−0.07	0.42	0.70	0.75	18.56	3.73	0.47	−10.91*
SE3	7.00	19.05	19.00	3.84	−0.15	−0.14	0.48	0.76	0.76	18.72	3.98	0.48	2.86
SE4	5.00	11.53	12.00	2.49	0.18	0.02	0.29	0.52	0.64	12.43	2.93	0.41	−12.06*
SE5	6.00	14.49	14.00	2.62	−0.02	0.11	0.26	0.51	0.70	14.65	3.4	0.44	−0.91
SE6	6.00	10.53	10.00	3.20	0.59	−0.11	0.48	0.75	0.74	12.95	3.69	0.48	−25.07*
SE total	37.00	87.22	87.00	13.64	0.03	0.01	0.38	0.87	0.91	92.58	15.79	0.44	−13.03*
BM1	6.00	12.19	12.00	3.35	0.48	−0.02	0.48	0.74	0.78	12.21	3.38	0.53	−0.17
BM2	8.00	16.76	16.00	4.73	0.50	−0.30	0.55	0.82	0.85	17.56	4.89	0.59	−5.61*
BM3	5.00	8.72	8.00	2.95	0.79	0.25	0.53	0.76	0.73	9.91	2.78	0.49	−13.35*
BM4	7.00	14.58	14.00	4.14	0.43	−0.06	0.55	0.81	0.81	14.71	4.04	0.55	−1.08
BM5	5.00	8.83	8.00	2.95	0.71	0.07	0.49	0.73	0.71	9.49	2.88	0.47	−7.38*
BM total	31.00	61.09	59.00	15.83	0.60	0.03	0.52	0.94	0.93	63.89	15.35	0.56	−5.88*

*N = 1101. Ni, number of items per facet or scale; Sk, skewness; Ku, kurtosis; α, Cronbach Alpha; CITC, corrected item to total correlation. t (1100) = mean comparison between construction sample (German language version) and current sample. p < 0.01 (Bonferroni corrected)*.

The internal consistencies for the total scores of cheerfulness, seriousness, and bad mood were high (CH α = 0.89; SE α = 0.87; BM α = 0.94) and comparable to the internal consistencies of the German version of the STCI-T <106> and an American sample that had completed an English pilot version (CH α = 0.93; SE α = 0.89; BM α = 0.92; see Ruch and Carrell, 1997). With respect to the facets, the five facets of trait cheerfulness all yielded satisfactory reliabilities ranging from α = 0.72 to α = 0.88, apart from facet CH3, with an α = 0.65, see Table 3. Looking at the facets of trait seriousness, the internal consistencies ranged from α = 0.51 to α = 0.76. The facets of bad mood all reached satisfactory internal consistencies, between α = 0.73 and α = 0.82. Overall, the internal consistencies of the facets were highly comparable to the scores reported for the German version of the STCI-T <106> (see Ruch et al., 1996), apart from the facet SE5, which yielded a lower α in the English version (α = 0.51 compared to α = 0.70 in the German version), see Table 3. The skewness values (ranging between −0.66 to 0.79) were numerically comparable to the German language version questionnaire and the kurtosis values (ranging −0.30 to 0.25) were numerically slightly lower as compared to the German language version.

When looking at the items of the facets of the three traits, the corrected item to total correlations (CITC) were generally satisfactory to high. For the facets of trait cheerfulness, the mean CITC for the items of a facet ranged between 0.34 to 0.66 (CH1: *r*_m_ = 0.66; CH2: *r*_m_ = 0.55; CH3: *r*_m_ = 0.34; CH4: *r*_m_ = 0.43; CH5: *r*_m_ = 0.52; see Table 3), with six items having a low CITC (i.e.,>0.30). For the facets of trait seriousness, the mean CITC for the items of a facet ranged between 0.26 to 0.48 (SE1: *r*_m_ = 0.32; SE2: *r*_m_ = 0.42; SE3: *r*_m_ = 0.48; SE4: *r*_m_ = 0.29; SE5: *r*_m_ = 0.26; SE6: *r*_m_ = 0.48), with six items not reaching a minimal CITC of > 0.30. Lastly, with respect to the facets of trait bad mood, the mean CITC for the items of a facet ranged between 0.48 to 0.55 (BM1: *r*_m_ = 0.48; BM2: *r*_m_ = 0.55; BM3: *r*_m_ = 0.53; BM4: *r*_m_ = 0.55; BM5: *r*_m_ = 0.49), with one item not reaching a minimal CITC of > 0.30. Overall, the mean CITC were highly comparable to the ones reported for the German version of the STCI-T <106>, apart from a lower score for SE4 in the English language version, see Table 3.

### Mean comparisons and correlations

Next, we compared the means of the current English version to the means of the German construction sample (*t*-tests). Table 3 shows that the means generally differed between the two language versions in nearly all of the facets. With respect to the total scores, the means in the English language version indicated that the sample reported to higher scores in trait cheerfulness, less trait seriousness and less trait bad mood. Moreover, we computed Pearson correlations between the total scores and the facets of the three traits. The correlations can be seen in Table 4.

**Table 4:** Correlations of the facets and the total scores of the STCI–T <106> in the construction sample.

	**CH total**	**CH1**	**CH2**	**CH3**	**CH4**	**CH5**	**SE total**	**SE1**	**SE2**	**SE3**	**SE4**	**SE5**	**SE6**	**BM total**	**BM1**	**BM2**	**BM3**	**BM4**	**BM5**
CH total	1	0.89^***^	0.85^***^	0.79^***^	0.84^***^	0.89^***^	−0.46^***^	−0.40^***^	−0.37^***^	−0.17^***^	−0.29^***^	−0.19^***^	−0.62^***^	−0.73^***^	−0.64^***^	−0.60^***^	−0.72^***^	−0.61^***^	−0.65^***^
CH1		1	0.72^***^	0.67^***^	0.62^***^	0.71^***^	−0.36^***^	−0.33^***^	−0.32^***^	−0.13^***^	−0.19^***^	−0.13^***^	−0.48^***^	−0.71^***^	−0.65^***^	−0.60^***^	−0.66^***^	−0.60^***^	−0.58^***^
CH2			1	0.56^***^	0.64^***^	0.74^***^	−0.37^***^	−0.29^***^	−0.31^***^	−0.13^***^	−0.24^***^	−0.15^***^	−0.52^***^	−0.59^***^	−0.52^***^	−0.44^***^	−0.61^***^	−0.48^***^	−0.57^***^
CH3				1	0.59^***^	0.59^***^	−0.33^***^	−0.31^***^	−0.34^***^	−0.16^***^	−0.16^***^	−0.08	−0.39^***^	−0.57^***^	−0.50^***^	−0.52^***^	−0.54^***^	−0.48^***^	−0.46^***^
CH4					1	0.75^***^	−0.47^***^	−0.39^***^	−0.32^***^	−0.18^***^	−0.36^***^	−0.26^***^	−0.62^***^	−0.58^***^	−0.49^***^	−0.47^***^	−0.62^***^	−0.46^***^	−0.55^***^
CH5						1	−0.44^***^	−0.39^***^	−0.31^***^	−0.13^***^	−0.29^***^	−0.19^***^	−0.64^***^	−0.63^***^	−0.53^***^	−0.49^***^	−0.65^***^	−0.53^***^	−0.61^***^
SE total							1	0.75^***^	0.80^***^	0.75^***^	0.76^***^	0.65^***^	0.71^***^	0.54^***^	0.42^***^	0.42^***^	0.49^***^	0.50^***^	0.55^***^
SE1								1	0.55^***^	0.46^***^	0.45^***^	0.35^***^	0.53^***^	0.47^***^	0.39^***^	0.40^***^	0.41^***^	0.43^***^	0.44^***^
SE2									1	0.55^***^	0.51^***^	0.44^***^	0.44^***^	0.50^***^	0.42^***^	0.43^***^	0.40^***^	0.47^***^	0.45^***^
SE3										1	0.51^***^	0.34^***^	0.30^***^	0.20^***^	0.14^***^	0.17^***^	0.18^***^	0.18^***^	0.21^***^
SE4											1	0.47^***^	0.52^***^	0.32^***^	0.23^***^	0.21^***^	0.33^***^	0.29^***^	0.39^***^
SE5												1	0.38^***^	0.28^***^	0.21^***^	0.20^***^	0.25^***^	0.28^***^	0.30^***^
SE6													1	0.63^***^	0.49^***^	0.45^***^	0.62^***^	0.56^***^	0.67^***^
BM total														1	0.88^***^	0.88^***^	0.87^***^	0.90^***^	0.82^***^
BM1															1	0.72^***^	0.70^***^	0.77^***^	0.66^***^
BM2																1	0.71^***^	0.72^***^	0.59^***^
BM3																	1	0.70^***^	0.73^***^
BM4																		1	0.71^***^
BM5																			1

*p < 0.05. **p < 0.01.

*** *p < 0.001*.

As expected, the numerically highest correlations were found for the facets of each respective trait with the total score (see Table 4). Also, correlations of homologous scales were higher than correlations to heterologous scales. In line with former findings, cheerfulness correlated negatively with seriousness and trait bad mood, with the latter two being positively correlated. Overall, the correlations replicated the patterns reported for the German and Spanish language version of the STCI-T (see Ruch et al., 1996; Carretero-Dios et al., 2014).

### Testing the underlying structure of the STCI-T <106>

Six alternative models regarding the disposition of the facet model were tested by structural equation modeling: a confirmatory factor analysis (CFA; in SPSS AMOS 20) was based on the STCI facets theoretically derived for cheerfulness (CH), seriousness (SE), and bad mood (BM), and empirically isolated by exploratory factorial analysis (Ruch et al., 1996). Alternative models on the disposition of the facet model were also tested, referring to different postulates of cheerfulness. For example, Schneider (1950) hypothesized that cheerfulness and bad mood form a bipolar dimension. If this held true, two factors would be extracted: One seriousness factor and a bipolar cheerfulness- bad mood factor (model 2).

The following models were tested:

Model 1, one factor: all facets on a general factor (CH-SE-BM);
Model 2, two factors: cheerfulness-bad mood (CH-BM); and seriousness (SE);
Model 3, two factors: cheerfulness-seriousness (CH-SE); and bad mood (BM);
Model 4, two factors; cheerfulness (CH); and seriousness-bad mood (SE-BM);
Model 5, three factors: cheerfulness (CH); bad mood (BM) and seriousness (SE);
Model 6, three factors: cheerfulness (CH); bad mood (BM) and seriousness (SE); and second loadings for several facets (CH1 and CH3 on BM; SE6 on CH; BM3 and BM5 on CH).

The CFA analysis was based on a correlation matrix and maximum likelihood estimation (ML) was employed. A multifaceted approach was used to evaluate the model fit (see Tanaka, 1993; Hu and Bentler, 1999). The reported chi-square denominates the difference between the observed data and the data implied by the specified model. Yet, the chi-square test usually produces a significant value in large samples (*N* > 1,000), even if the difference between the observed and implied data is trivial. Several goodness-of-fit indices were used to evaluate the models, including the root-mean-square error of approximation (RMSEA), standardized root mean square residual (SRMR), normed fit index (NFI), and Tucker-Lewis coefficient (TL, known as well like non-normed fit index). In general, it is considered that a fit index above 0.90 for NFI and TL as well as RMSE and SRMR values lower 0.1, are indicators of an acceptable fit (Bollen and Long, 1993; Browne and Cudeck, 1993). Cut off values of 0.95 or higher for NFI and TL, and of 0.05 or lower for RMSEA and SRMR signify a good model fit (Hu and Bentler, 1999). Before performing the analysis, descriptive statistics were checked (see Table 3). Table 3 shows that none of the facets deviated from normal distribution. Average absolute levels of the skewness and kurtosis of the facets were in the acceptable range for structural equation model analysis using likelihood estimation (Muthen and Kaplan, 1985), see Table 3.

For the exploratory analysis, the Kaiser-Meyer-Olkin (KMO) and Bartlett's sphericity tested the sampling adequacy for applying factorial analysis. KMO value was 0.93, and the Bartlett's test showed statistical significance (χ^2^ = 12041.43, *df* = 120, *p* < 0.001), indicating that the samples met the criteria for factor analysis. A principal axis analysis performed on the facet inter-correlations revealed three factors exceeding unity (Eigenvalues were 7.83, 2.12, 1.19, 0.84, 0.62, 0.58, and 0.44) and also the Scree-test suggested the retention of three factors, which explained 69.05% of the variance. Moreover, we computed a parallel analysis (Horn, 1965) to verify the retention of the three factors (using the SPSS syntax provided by O'Connor, 2000). In this analysis, the eigenvalues obtained in the dataset were compared to generated eigenvalues from PAF of 100 datasets (random data generated by permutations of the original raw dataset). The first three eigenvalues met the criterion for retention (i.e., their mean exceeded the randomly generated mean across 100 datasets, with the first four random means being 1.21, 1.16, 1.13, 1.10) and thus exceeding the upper 95th percentile of the distribution of the eigenvalues retrieved from the 100 random datasets. The location of the centroids indicated that the concepts were not orthogonal. An oblique rotation was undertaken, and the reference structure of the factors is given in Table 5.

**Table 5:** Loadings of the STCI–T <106> facets on the three unrotated and three obliquely rotated factors in the construction sample.

**Facets**	**F1**	**F2**	**F3**	**Obl 1**	**Obl 2**	**Obl 3**	**h^2^**
**CHEERFULNESS**							
CH1	−0.78	0.31	0.09	–*0.63*	0.12	−0.33	0.72
CH2	−0.73	0.25	0.28	–*0.80*	0.04	−0.04	0.67
CH3	−0.65	0.23	0.10	–*0.54*	0.05	−0.23	0.49
CH4	−0.75	0.10	0.34	–*0.82*	−0.13	0.06	0.69
CH5	−0.80	0.21	0.37	–*0.92*	−0.03	0.04	0.82
**SERIOUSNESS**							
SE1	0.48	0.39	−0.06	0.13	*0.55*	0.03	0.39
SE2	0.58	0.47	0.14	−0.09	*0.64*	0.30	0.58
SE3	0.33	0.58	−0.03	−0.07	*0.70*	−0.04	0.44
SE4	0.47	0.58	−0.12	0.12	*0.76*	−0.10	0.58
SE5	0.37	0.47	0.01	−0.04	*0.59*	0.04	0.36
SE6	0.75	0.19	−0.14	0.45	*0.41*	0.11	0.62
**BAD MOOD**							
BM1	0.78	−0.18	0.35	0.06	−0.02	*0.84*	0.76
BM2	0.74	−0.15	0.35	0.02	0.00	*0.82*	0.68
BM3	0.84	−0.14	0.11	0.34	0.07	*0.55*	0.73
BM4	0.79	−0.08	0.38	−0.02	0.09	*0.86*	0.78
BM5	0.80	−0.03	0.09	0.29	0.20	*0.48*	0.65

*N = 1101. Expected loadings were italicized. F, unrotated factors; Obl, rotated factors; h^2^, communality*.

The factors were identified as cheerfulness (1), seriousness (3) and bad mood (2). Each facet loaded highest on the factor it belongs to. However, it was also observed that important second loadings appeared for CH1, CH3, and SE2 on the bad mood factor, and SE6, BM3, and BM5 loaded also on cheerfulness. The loading of SE6 on cheerfulness (−0.45) exceeded the one obtained for the factor of seriousness (0.41). The inter-correlations among the factors showed that the cheerfulness factor correlated mildly negatively with seriousness (*r* = −0.46, *p* < 0.001) and highly negatively with the bad mood factor (*r* = −0.73, *p* < 0.001), and the two forms of humorlessness were positively correlated (*r* = 0.54, *p* < 0.001). Next, confirmatory factor analysis was performed on the facets (ML estimation). The measures of fit obtained with the different models are shown in Table 6.

**Table 6:** Assessment of fit of the STCI–T <106> data.

**Models**	**Chi–square**	***df***	**RMSEA**	**SRMR**	**NFI**	**TL**
Model 1	4696.13	104	0.21	0.11	0.73	0.70
Model 2	3089.60	103	0.16	0.10	0.81	0.79
Model 3	3756.63	103	0.18	0.11	0.78	0.76
Model 4	3490.43	103	0.17	0.10	0.79	0.77
Model 5	1829.67	101	0.10	0.08	0.88	0.86
Model 6	1180.47	96	0.09	0.06	0.92	0.90

*N = 1101. RMSEA, root–mean–square error of approximation; SRMR, standardized root mean square residual; NFI, Normed Fit Index; TL, Tucker–Lewis coefficient*.

Table 6 shows that model 1 (all facets on a general factor) yielded the worst fit. Although all two factor models (model 2: cheerfulness-bad mood and seriousness; model 3: cheerfulness-seriousness and bad mood; model 4: cheerfulness and seriousness-bad mood) showed a poor fit and none of goodness-of-fit indices considered were over the limit of a reasonable fit, it should be pointed out that the model 2 presented the best fit of these two-factorial options (which would be in line with Schneider, 1950). Third, model 5 (three factors: cheerfulness, seriousness, and bad mood) showed an acceptable fit index, with a RMSEA of 0.1 and a SRMR of 0.08. Nevertheless, the NFI and Tucker-Lewis coefficient indicated the fit of the model to the data was not acceptable (NFI = 0.88; TL = 0.89). Finally, the best fit was observed for model 6. Although the fit of the expected model (model 6) was not exceptionally good, a TL of 0.90 and a NFI of 0.92, were acceptable fit indices, and in line with Bollen and Long (1993), a RMSEA of 0.09 SRMR of 0.06 would denominate the limit of a reasonable error. The inspection of residuals showed that the fit for model 6 would improve if residuals were allowed to correlate (particularly the one among the facets of each factor). This result converges with previous research at item level, and reflects that facets forming each scale are not logically independent from each other (due to the antithetical nature of the traits). Additionally, higher modification indices would appear if a relation between SE3 and bad mood, or between SE3 and cheerfulness would be introduced. The standardized pattern coefficients obtained for model 6 are shown in Table 7.

**Table 7:** Standardized coefficients for Model 6.

**Facets**	**Cheerfulness**	**Seriousness**	**Bad mood**
CH1	0.59		−0.30
CH2	0.82		
CH3	0.54		−0.20
CH4	0.82		
CH5	0.90		
SE1		0.63	
SE2		0.76	
SE3		0.65	
SE4		0.72	
SE5		0.59	
SE6	−0.50	0.43	
BM1			0.87
BM2			0.83
BM3	−0.33		0.60
BM4			0.88
BM5	−0.30		0.57

*Model 6: three factors, (CH); Bad Mood (BM), and Seriousness (SE); and second loadings for several facets (CH1 and CH3 in BM; SE6 in CH; and BM3 and BM5 in CH)*.

The coefficients shown in Table 7 could be taken as indices of the precision with which the corresponding facets measures the factor and these correspond to the reliability analysis presented in Table 3, reinforcing confidence in the model 6 estimations. The standardized coefficients ranged from 0.59 (CH1) to 0.90 (CH5) for cheerfulness; from 0.43 (SE6) to 0.76 (SE2) for seriousness; and from 0.57 (BM5) to 0.88 (BM4) for bad mood. The second loadings ranged from −0.29 (CH3 in bad mood) to −0.50 (SE6 in cheerfulness).

### Adaptation of the standard trait form STCI-T <60> in the construction sample

Next, we aimed at deriving a 60 item standard form of the STCI-T <106> long form, parallel to the standard form in German and other languages. Following criteria were applied for the item selection: (a) the best corrected item to total correlation (CITC) with the own scale, (b) consideration of items content in order to preserve the content domains, (c) balanced representation of the facets (if impossible, core facets got more weight), and (d) avoidance of items with similar content or linguistic usage, (e) a good convergence with the item content of the German version (i.e., if item characteristics were similar, the paralleled item was chosen), and (f) there should be 20 items per scale. In general, a concept-guided strategy in item reduction was preferred to a purely empirical selection of items (as for the German standard form, see Ruch et al. (1996), although indices derived from PAF and item analyses were considered. Descriptive statistics (mean, standard deviation, skewness, and kurtosis), CITC and Cronbach Alpha (α) of the STCI-T <60> are given in Table 8.

**Table 8:** Descriptive statistics, corrected item to total correlation and reliability of the standard English version STCI–T <60> in the construction sample.

**Scale**	***Ni***	***M***	***SD***	***Sk***	***Ku***	**α**	**CITC**		
							***M***	**Min**	**Max**
CH	20	64.39	9.78	−0.49	−0.01	0.93	0.61	0.39	0.75
SE	20	50.10	8.37	−0.16	0.03	0.84	0.42	0.25	0.52
BM	20	39.28	11.04	0.57	0.02	0.92	0.59	0.42	0.72

*N = 1101. CH, cheerfulness; SE, seriousness; BM, bad mood; Ni, number of items per facet; Sk, skewness; Ku, kurtosis; α, Cronbach Alpha; CITC, corrected item to total correlation*.

Table 8 shows that none of the factors deviated from normal distribution. Cronbach Alpha ranged from α = 0.84 (seriousness) to α = 0.93 (cheerfulness) With respect to the CITC, all correlations were as expected, with means of the CITC being *r*_m_ = 0.61 for cheerfulness, *r*_m_ = 0.42 for seriousness, and *r*_m_ = 0.59 for bad mood, ranging from *r* = 0.39 to *r* = 0.75 for the items of cheerfulness to the cheerfulness scale, *r* = 0.25 to *r* = 0.52 for seriousness and *r* = 0.42 to *r* = 0.72 for bad mood. With respect to the correlations of the items with the other scales, all correlations were numerically lower than the CITC correlations and in the expected direction (*r* = −0.61 to *r* = −0.28 and *r* = −35 to *r* = 0.00 for the bad mood and seriousness items to the cheerfulness scale respectively; *r* = −0.33 to *r* = −0.01 and *r* = 0.28 to *r* = 0.13 for the cheerfulness and bad mood items to the seriousness scale respectively; *r* = −0.62 to *r* = −0.31 and *r* = 0.01 to *r* = 0.40 for the cheerfulness and seriousness items to the bad mood scale respectively).

### Structure of the STCI-T <60>

Next, we checked the structure of the STCI-T <60> by means of factor analysis. As the STCI-T utilizes a four-point Likert format, several problems may arise when using confirmatory factor analysis at item level. It is recommended to consider Likert responses as continuous variables without normal distribution (Bentler, 1995), and work on the asymptotic matrix of covariance in order to estimate the fit. However, this model makes strong assumptions which are difficult to verify. Furthermore, it requires very large samples to obtain accurate results in large models (*N* > 20 items). For this reason, we conducted an exploratory factor analysis in MPlus (6.11; Muthén and Muthén, 2005) with a robust least squares estimator (WLSMV) and by means of using polychoric correlations to analyze the STCI-T <60>. The main goal was to have the most parsimonious solution that represents the data well. Consequently, models with one to five factors were compared and several fit indices were used to evaluate the model fit (CFI ≥0.90; RMSEA/SRMR ≤ 0.8; Browne and Cudeck, 1993; Hu and Bentler, 1999)≥0.9. Table 9 shows the Chi square values, CFI, RMSEA, and SRMR for the five different factor solutions.

**Table 9:** Exploratory factor analysis on the STCI–T <60> in the construction sample.

**Models**	**Chi–square**	***df***	***p***	**CFI**	**RMSEA**	**SRMR**
1 Factor	13482.96	1710	<0.001	0.824	0.079	0.093
2 Factor	7655.97	1651	<0.001	0.907	0.057	0.058
3 Factor	4382.69	1593	<0.001	0.957	0.040	0.039
4 Factor	3394.04	1536	<0.001	0.967	0.033	0.032
5 Factor	2967.52	1480	<0.001	0.972	0.030	0.029

As a result of the factor analysis, the first seven Eigenvalues were: 19.23, 5.14, 3.07, 1.88, 1.42, 1.23, and 1.24. As Table 9 shows, the CFI increases from a one to a three factor solution (with the CFI of the three factor solution meeting the criterion) and does not increase much more in a four factor solution. Thus, the extraction of a forth factor would not lead to a big increase in the fit indices. Therefore, three factors were extracted and rotated obliquely (Oblimin-criterion; delta = 0; see Table 10).

**Table 10:** Loadings of the STCI–T <60> items on the three obliquely rotated factors in the construction sample.

**Items**	**Obl 1**	**Obl 2**	**Obl 3**	**Items**	**Obl 1**	**Obl 2**	**Obl 3**	**Items**	**Obl 1**	**Obl 2**	**Obl 3**
1BM	*0.48*			2CH		*0.43*		3SE			*0.37*
6BM	*0.57*			4CH		*0.69*		5SE			*0.36*
11BM	*0.54*			9CH		*0.85*		7SE			*0.39*
8BM	*0.75*			14CH		*0.56*		12SE			*0.65*
13BM	*0.77*			16CH		*0.68*		10SE	0.23		*0.23*
17BM	*0.66*			19CH		*0.74*		20SE			*0.39*
21BM	*0.36*	0.22		22CH		*0.79*		18SE			*0.35*
24BM	*0.48*			57CH		*0.70*	−0.22	23SE			*0.64*
40BM	*0.73*			25CH		*0.72*		28SE			*0.49*
27BM	*0.65*			26CH		*0.71*		33SE			*0.52*
31BM	*0.55*	0.26		30CH		*0.86*		36SE			*0.31*
29BM	*0.76*			32CH		*0.74*		42SE	0.25		*0.38*
34BM	*0.76*			41CH		*0.69*		39SE			*0.57*
43BM	*0.78*			38CH		*0.66*		47SE			*0.62*
37BM	*0.71*	0.21		35CH		*0.44*		52SE			*0.55*
45BM	*0.61*			46CH	−0.26	*0.66*		15SE			*0.50*
54BM	*0.64*	0.25		44CH		*0.58*		55SE			*0.49*
48BM	*0.71*			50CH		*0.71*		60SE			*0.58*
51BM	*0.40*	0.21		53CH		*0.60*		58SE			*0.50*
56BM	*0.64*			59CH		*0.66*		49SE	0.25		*0.51*

*N = 1101. The items are grouped by factor. CH, cheerfulness; SE, seriousness; BM, bad mood. Expected loadings were italicized. Obl, rotated factors. All listed loadings > 0.20*.

The three factors were clearly identified as the three theoretically expected factors of cheerfulness (1), seriousness (2), and bad mood (3), in line with other recent findings on the pilot version of the STCI-T <60> (see Lau et al., 2018). All items loaded highest on their theoretically expected factor and no important second loadings occurred, see Table 10. The size of the intercorrelations for CH vs. SE (*r* = −0.28, *p* < 0.01) and SE vs. BM (*r* = 0.28, *p* < 0.001) was reduced compared with to the STCI-T <106>. The correlation for CH vs. BM (*r* = −0.65, *p* < 0.001) was similar to the STCI-T <106>. The STCI-T <60> can be seen in Appendix A.

## Psychometric characteristics of the STCI-T <60> in english in the replication sample

Next, we checked the scale characteristic of the STCI-T <60> in an independently collected sample where also peer-reports were available. The scale characteristics of the STCI-T <60> in self-and peer report can be seen in Table 11.

**Table 11:** Descriptive statistics, corrected item to total correlation and reliability of the standard English version STCI–T <60> (self– and peer–reports) in the replication sample and peer–report sample.

								**CITC**		
	***M***	***SD***	***Sk***	***K***	**Min**	**Max**	**α**	**Min**	**Max**	***M***
**SELF–REPORT**										
Cheerfulness	65.46	10.07	−0.59	−0.01	35	80	0.94	0.44	0.77	0.63
Seriousness	57.86	8.42	0.21	−0.43	39	77	0.84	0.21	0.61	0.42
Bad mood	35.13	10.71	0.58	−0.45	20	61	0.92	0.42	0.74	0.59
**PEER–REPORT**										
Cheerfulness	66.02	11.04	−0.79	−0.31	39	80	0.95	0.10	0.76	0.46
Seriousness	59.15	10.47	−0.43	0.28	30	80	0.85	0.16	0.63	0.22
Bad mood	36.14	13.58	0.98	0.21	20	73	0.94	0.08	0.78	0.43

*N = 79–87. Sk, skewness; Ku, kurtosis; α, Cronbach Alpha; CITC, corrected item to total correlation; Each facet contains 20 items*.

Table 11 shows that the descriptive statistics and the high reliabilities of the STCI-T <60> were replicated for the self-reports in the Replication Sample and were also high in the peer-report version (Peer Report Sample). The CITC were sufficient, with only few exceptions, and generally replicating the results from the Construction Sample. When looking at the means of the self-reports as compared to the peer-reports, no significant differences were detected (*p*-values ranging from 0.10 to 0.93, all n.s.) between self-and peer-reports. Moreover, the correlations between the self- and peer-reported traits indicated a moderate to good convergence (*r* = 0.74 for CH, *r* = 0.33 for SE and *r* = 0.65 for BM, all *p* < 0.001).

## Discussion

The aim of the current article was to test the factorial structure of the STCI-T <106> and provide the psychometric characteristics in the English language version, as well as the adaptation of the short form with 60 items. Most importantly, the postulated facet structure of the STCI-T <106> has been confirmed in the English language version. Using a structural equation model approach, six alternative models on the disposition of the facet model were tested in the Construction Sample. Results confirmed that the theoretically derived model with three factors agreed acceptably well with the data, and presented the best fit among the tested models. Specifically, this model consisted in three related factors of cheerfulness, seriousness and bad mood, with second theoretically derived loadings for CHl (prevalence of cheerful mood) and CH3 (composed view of adverse life circumstances) on bad mood factor; SE6 (a humorless attitude about cheerfulness-related matters) on cheerfulness; and BM3 (sad individual's prototypical behavior in cheerfulness evoking situations) and BM5 (ill-humored individual's prototypical behavior in cheerfulness evoking situations) on cheerfulness.

Moreover, in line with the expectations, the psychometric characteristics of the STCI-T <106> were sufficient to good and comparable with the German parent version. All scales and subscales were normally distributed and had an adequate internal consistency. However, facets SE1, SE4, and SE5 presented a low internal consistency, diverging from internal consistency values reported by Ruch and colleagues (they were higher: SE1 α = 0.65; SE4 α = 0.64; SE5 α = 0.70; Ruch et al., 1996). This may be explained by the restricted variance of seriousness in the Construction Sample (i.e., under-representation of individuals over 40 years of age). In the present sample, participants aged from 15 to 70 years (*M* = 24.85; *SD* = 10.11), whereas in the German sample used to develop the STCI-T participants aged from 14 to 83 (*M* = 33.90; *SD* = 15.09), being older on average. Therefore, further research is needed to investigate the reliability of the STCI-T facets for the 106 item long form. Yet, the lower reliabilities are not problematic for the current version when being used in research. Also, it is not suggested to use the facets for diagnostic purposes, but refer to the total scores (which all show high reliabilities). Moreover, we found mean differences between the German and English samples, indicating that English participants were more cheerful and less serious, and less habitually in a bad mood as compared to German participants. To summarize, the English language version of the STCI-T <106> shows sufficient item and scale characteristics and good reliabilities for the total scores, comparable to the German version. Future studies will need to focus on replicating and providing more evidence of the scales' external validity and criterion validity for the English language version.

After looking at the characteristics of the long form of the STCI, we also adapted the standard form with 60 items in two samples. Most importantly, the item and scale characteristics were as expected and comparable to the German language version. Also, additional peer-reports showed good comparability of self- and peer-reports on cheerfulness, seriousness and bad mood.

The current study has several limitations. First, following the guidelines by Smith et al. (2000) for short form constructions, the STCI-T <60> still needs to be validated in independently collected sample that allows performing confirmatory factor analyses (i.e., having a sufficiently large *N*). This could help showing that the parent form and the short form show a good congruence of factor solutions. Second, future studies should include a sample of individuals that complete the long, as well as the short form. Third, the translation procedure utilized in this approach deviated from a classical translation-back translation procedure. Forth, future studies will need to investigate the suitableness of this English language version across nations (USA, UK, Australia, etc.). Despite those limitations, the English STCI is ready for use in research. Whereas the long form (106 items) allows a fine-grained analysis of the facets, the standard form with 60 items serves as a more economic assessment tool of cheerfulness, seriousness, and bad mood.

To conclude, the STCI-T <106>, as well as the short form with 60 items (STCI-t <60>) are ready for further validations and use. Future studies should aim at investigating the incremental validity of the *State-Trait Cheerfulness Inventory* in the prediction of humor related outcomes when controlling for broader personality traits (i.e., the “Big Five,” especially extraversion). Also, future studies should investigate the relationship of different models describing the sense of humor and related traits (such as playfulness, see Proyer, 2012, 2018), as well as looking more deeply into cheerfulness interventions (see Papousek and Schulter, 2008, 2010). Moreover, future studies may opt for more balanced samples in terms of gender ratio.

## Author's note

JH is at the Department of Psychology at the University of Zurich, Switzerland. HC-D is at the Department of Research Methods in Behavioral Sciences, University of Granada, Spain. AC is at the Department of English at the University of Central Oklahoma, Edmond, United States of America. Our thanks go to Lambert Deckers and Willibald Ruch for helping with the data collection and Willibald Ruch for providing helpful comments on a prior version of the manuscript.

## Author contributions

JH, HC-D: concept; AC: data collection; JH, HC-D: analyses; JH, HC-D: writing draft; JH, AC, HC-D: revisions and feedback.

### Conflict of interest statement

The authors declare that the research was conducted in the absence of any commercial or financial relationships that could be construed as a potential conflict of interest.
