# Supplementary material for: Assessing the Temperamental Basis of the Sense of Humor: Adaptation of the English Language Version of the State-Trait Cheerfulness Inventory Long and Standard Form
Source: Front Psychol. 2018 Nov 27;9:2255. doi: 10.3389/fpsyg.2018.02255 (PMC6277566; doi:10.3389/fpsyg.2018.02255)
Supplement: Supplementary file 1 [file Table_1.DOC]

Instructions:

The following statements refer to your moods and mentality **in general**. Please try as much as possible to describe your **habitual** behavior patterns and attitudes by marking an X through one of the four alternatives. Please use the following scale:

(1) strongly disagree

(2) moderately disagree

(3) moderately agree

(4) strongly agree

For example:

I am an active person. (1) (2) (3) (4)

If you strongly agree with this statement, that is, if you are **in general** an active person, **mark an X through (4)**. If you strongly disagree, that is, if you are **habitually** ***not*** active ***at all***, **mark an X through (1)**. If you have difficulty answering a question, pick the response that **most** applies.

Please answer *every* question, do not omit any.

| 1 | People often have reason to ask if something is eating me. | (1) (2) (3) (4) |
| --- | --- | --- |
| 2 | My way of life can be described as positive and carefree. | (1) (2) (3) (4) |
| 3 | I very seldom act without a proper reason. | (1) (2) (3) (4) |
| 4 | I am a cheerful person. | (1) (2) (3) (4) |
| 5 | Most of my friends are more likely to be serious and reflective. | (1) (2) (3) (4) |
| 6 | Some annoying circumstances are capable of spoiling my mood for quite a while. | (1) (2) (3) (4) |
| 7 | I prefer conversations that deal with important things and are very profound. | (1) (2) (3) (4) |
| 8 | Sometimes I have the feeling of an inner emptiness. | (1) (2) (3) (4) |
| 9 | I can be made to laugh easily. | (1) (2) (3) (4) |
| 10 | I find it unnecessary when people exaggerate in talking to me. | (1) (2) (3) (4) |
| 11 | Compared to others, I really can be grumpy and grouchy. | (1) (2) (3) (4) |
| 12 | I plan my actions and make my decisions so that they are useful to me in the long run. | (1) (2) (3) (4) |
| 13 | I often feel despondent. | (1) (2) (3) (4) |
| 14 | I can easily unwind and enjoy the moment. | (1) (2) (3) (4) |
| 15 | I am a serious person. | (1) (2) (3) (4) |
| 16 | Everyday life often gives me the occasion to laugh. | (1) (2) (3) (4) |
| 17 | I often think, "For heaven's sake, don't bother me today.” | (1) (2) (3) (4) |
| 18 | In my life, I like to have everything correct. | (1) (2) (3) (4) |
| 19 | I have a "sunny" nature. | (1) (2) (3) (4) |
| 20 | When I watch TV, I prefer informative reports to "shallow" programs. | (1) (2) (3) (4) |
| 21 | When I am distressed, even a very funny thing fails to cheer me up. | (1) (2) (3) (4) |
| 22 | I often smile. | (1) (2) (3) (4) |
| 23 | In everything I do, I always consider every possible effect and compare all pros and cons carefully. | (1) (2) (3) (4) |
| 24 | When friends try to cheer me up by joking or fooling around, I sometimes become more morose and grumpy. | (1) (2) (3) (4) |
| 25 | Laughing has a contagious effect on me. | (1) (2) (3) (4) |
| 26 | I often find that the small things in everyday life are really funny and amusing. | (1) (2) (3) (4) |
| 27 | There are many days on which I think, "I got up on the wrong side of bed." | (1) (2) (3) (4) |
| 28 | In most situations, I initially see the serious aspect. | (1) (2) (3) (4) |
| 29 | Sometimes I am sad without any reason. | (1) (2) (3) (4) |
| 30 | I like to laugh and do it often. | (1) (2) (3) (4) |
| 31 | My mood is often not the best one. | (1) (2) (3) (4) |
| 32 | I am a merry person. | (1) (2) (3) (4) |
| 33 | When I am in contact with others, I often find that I have thought many things through more thoroughly than they. | (1) (2) (3) (4) |
| 34 | Even if there is no reason, I often feel ill-humored. | (1) (2) (3) (4) |
| 35 | Many adversities of everyday life actually do have a positive side. | (1) (2) (3) (4) |
| 36 | In conversation, I always avoid exaggerations, embellishments, and ambiguities, all of which do not contribute to the meaning of my statements. | (1) (2) (3) (4) |
| 37 | I am often in a bad mood. | (1) (2) (3) (4) |
| 38 | I feel completely contented being with cheerful people. | (1) (2) (3) (4) |
|  | Please turn the page. - | |
| 39 | My everyday life is filled mainly with important things and matters. | (1) (2) (3) (4) |
| 40 | Sometimes I am distressed for a very long time. | (1) (2) (3) (4) |
| 41 | The good mood of others has a contagious effect on me. | (1) (2) (3) (4) |
| 42 | I don't understand how others can waste their time on senseless matters. | (1) (2) (3) (4) |
| 43 | I am often sullen. | (1) (2) (3) (4) |
| 44 | I often find the slight mishaps of everyday life amusing, even  if they happen to me. | (1) (2) (3) (4) |
| 45 | My acquaintances often get on my nerves. | (1) (2) (3) (4) |
| 46 | I am often in a good mood, even without a specific reason. | (1) (2) (3) (4) |
| 47 | I tend to plan far in advance and to set long-term goals for myself. | (1) (2) (3) (4) |
| 48 | I often feel so gloomy that nothing can make me laugh. | (1) (2) (3) (4) |
| 49 | Even seemingly trivial things have to be treated seriously and responsibly. | (1) (2) (3) (4) |
| 50 | I am often in a joyous mood. | (1) (2) (3) (4) |
| 51 | If I am in a bad mood, I can't stand the presence of cheerful people. | (1) (2) (3) (4) |
| 52 | I try to spend my free time doing things as useful as possible. | (1) (2) (3) (4) |
| 53 | Experience has shown me that the proverb "Laughter is the best medicine" is really true. | (1) (2) (3) (4) |
| 54 | I am a rather sad person. | (1) (2) (3) (4) |
| 55 | I prefer people who communicate with deliberation and objectivity. | (1) (2) (3) (4) |
| 56 | I often feel so weary that I cannot rouse myself to do anything. | (1) (2) (3) (4) |
| 57 | I like to kid around with others. | (1) (2) (3) (4) |
| 58 | When I communicate with other people, I always try to have an objective and sober exchange of ideas. | (1) (2) (3) (4) |
| 59 | It is easy for me to spread good cheer. | (1) (2) (3) (4) |
| 60 | One of my principles is: "first work, then play.” | (1) (2) (3) (4) |

**Scoring key for the STCI-T<60>**

The standard version of the STCI-T (with 60 items) measures three dimensions: trait-cheerfulness (CH), trait-seriousness (SE), and trait-bad mood (BM).

The four answer alternatives are coded as follows: "strongly disagree" = 1, "moderately disagree" = 2, "moderately agree" = 3, and "strongly agree" = 4.

Below you find the formulas for the three scales.

CH: 2 + 4 + 9 + 14 + 16 + 19 + 22 + 25 + 26 + 30 + 32 + 35 + 38 + 41 + 44 + 46 + 50 + 53 + 57 + 59

SE: 3 + 5 + 7 + 10 + 12 + 15 + 18 + 20 + 23 + 28 + 33 + 36 + 39 + 42 + 47 + 49 + 52 + 55 + 58 + 60

BM: 1 + 6 + 8 + 11 + 13 + 17 + 21 + 24 + 27 + 29 + 31 + 34 + 37 + 40 + 43 + 45 + 48 + 51 + 54 + 56
